# Impaired mitochondria of Tregs decreases OXPHOS-derived ATP in primary immune thrombocytopenia with positive plasma pathogens detected by metagenomic sequencing

**DOI:** 10.1186/s40164-022-00304-y

**Published:** 2022-09-01

**Authors:** Yanxia Zhan, Jingjing Cao, Lili Ji, Miaomiao Zhang, Qi Shen, Pengcheng Xu, Xibing Zhuang, Shanshan Qin, Fanli Hua, Lihua Sun, Feng Li, Hao Chen, Yunfeng Cheng

**Affiliations:** 1 Department of Hematology, Zhongshan Hospital, Fudan University, 180 Fenglin Rd, Shanghai, 200032 China; 2 Institute of Clinical Science, Zhongshan Hospital, Fudan University, Shanghai, 200032 China; 3 Center for Tumor Diagnosis & Therapy, Jinshan Hospital, Fudan University, Shanghai, 201508 China; 4 Department of Hematology, Zhongshan Hospital Qingpu Branch, Fudan University, Shanghai, 201700 China; 5 Department of Thoracic Surgery, Zhongshan Hospital Xuhui Branch, Fudan University, 966 Mid Huaihai Rd, Shanghai, 200031 China

**Keywords:** Immune thrombocytopenia, Tregs (Regulatory T cells), Glycolysis, Oxidative phosphorylation, Pathogens

## Abstract

**Background:**

Primary immune thrombocytopenia (ITP) is an autoimmune disease. Some ITP patients are associated with pathogen infection undetected with conventional technologies. Investigating the changes of T cells and potential metabolic mechanism are important for better understanding of ITP.

**Methods:**

The study enrolled 75 newly diagnosed ITP patients. The pathogens of patients were detected by metagenomic next-generation sequencing (mNGS). Plasma lipids were measured by liquid chromatography-mass spectrometry (LC–MS). CD4 T cell and CD8 T cell were analyzed using flow cytometry. Mitochondrial reactive oxygen species (ROS) and mitochondrial membrane potential were measured by flow cytometry. Seahorse XF real-time ATP rate assay was used to investigate the change of cellular metabolism.

**Results:**

Positive plasma pathogens were detected in seven ITP patients. Of them, 5 (71.4%) positive pathogen-ITP patients were no response (NR) after first-line treatment with corticosteroids. Regulatory T cells (Tregs) increased significantly in positive pathogen-ITP patients compared to negative pathogen-ITP patients and healthy controls (HC). Mitochondrial membrane potential of Th17 and Tregs were decreased in positive pathogen-ITP and negative pathogen-ITP patients, compared to HC (all *p* < 0.05). The overall metabolism flux of positive pathogen-ITP patients was decreased, as compared to HC (*p* = 0.004), of them a higher proportion of glycolysis-derived ATP and a smaller proportion of oxidative phosphorylation (OXPHOS)-derived ATP were found in Tregs. The ATP rate index of Tregs was decreased significantly in positive pathogen-ITP patients compared to negative pathogen-ITP patients and HC (*p* < 0.05).

**Conclusions:**

Impaired mitochondria function of Tregs in positive pathogen-ITP patients caused a decrease of OXPHOS-derived ATP and overall metabolism flux that might be the cause of steroid resistance in ITP patients.

## Background

Primary immune thrombocytopenia (ITP), an autoimmune disease, is characterized by thrombocytopenia which affects almost 1:10000 people of the world [1, 2]. The epidemiological characteristics of ITP is heterogeneous and diverse, as well as its clinical presentations and responses to first-line treatment of corticosteroids. The mechanism of thrombocytopenia has been summarized as destruction and insufficient production of platelets. Immunological factors that contribute to the thrombocytopenia have been investigated extensively, including autoantibody, CD4 T cell, CD8 T cell, platelet desialylation, T follicular helper (TFH) cell, and immune microenvironment [3–8], yet remains incompletely understood. Infectious pathogens that trigger thrombocytopenia have been reported in various infections, such as human immunodeficiency virus (HIV), Helicobacter pylori (Hp), hepatitis C virus (HCV), and coronavirus disease-2019 (COVID-19) [9–12]. Other than that, infectious agents accompanying with adult ITP patients have been far less investigated, due to limited detection methodologies. Thus, comprehensively evaluating of infection status of ITP patients would provide a better mechanistic understanding of the disease.

Thousands of microorganisms have been known to infect human, however, identification of the infectious agents of many diseases remains challenging due to the low rate of detection by conventional diagnostic methods. Benefit from rapid technological advances, metagenomic next-generation sequencing (mNGS) has recently been applied to detect pathogens, which can identify pathogens directly from patients’ clinical samples [13]. Moreover, mNGS could simultaneously detect nearly all known pathogens unbiasedly. mNGS is especially suitable for detecting novel and rare infectious agents. However, there has been no such study to evaluate the infection in adults ITP patients using mNGS.

Under normal circumstances, human immune system maintains a dynamic balance. Human body keeps a state of mutualism with innocuous, commensal microorganism. However, upon encountering harmful pathogens, T cells proliferate and differentiate into specialized subsets which secrete specialized cytokines participate in the response to infection. There has been a growing awareness that the change of T cell function is dependent on metabolic reprogramming [14–16]. Each immune cell subset depends on specific metabolic pathway for their function. Glycolysis and mitochondrial oxidative phosphorylation (OXPHOS) are two major pathways for energy generation and play a key role in energy homeostasis. Resting T cells mainly based on mitochondrial oxidative metabolism with glycolysis, fatty acid oxidation and glutaminolysis at a low rate [17]. However, Activated T cells increase glycolysis and glutaminolysis but decrease mitochondrial fatty acid oxidation, fulfill increased energy demands, and conserve lipids for new membrane synthesis [18–20]. Changes of T cell function have been widely confirmed in many diseases, ranging from cancer to infection disease and autoimmune disease, such as ITP [21, 22]. Therefore, modifying the metabolic pathway to selectively target T cell subset paves new avenue for potential treatment of diseases. Given the importance of T cell metabolic, the study aimed to assess the pathogens status of ITP patients, and to investigate the changes of T cell and the potential metabolic mechanism between positive plasma pathogen and negative pathogen ITP patients.

## Materials and methods

### Patients and controls

Seventy-five patients with newly diagnosed primary ITP were enrolled in the study (47 females and 28 males). All the patients were recruited from Zhongshan Hospital of Fudan University (Shanghai, China) between May 2016 and March 2021. The diagnosis of primary ITP and the response to treatment were evaluated according to the criteria updated by an international working group [23]. According to the criteria, complete response (CR) was defined as a platelet count ≥ 100 × 10^9^/L. Response (R) was defined as a platelet count ≥ 30 × 10^9^/L but < 100 × 10^9^/L and without bleeding. No response (NR) was defined as a platelet count < 30 × 10^9^/L or less than twofold increase of baseline or bleeding. The bleeding scale of ITP patients was calculated by the sum of age scale and bleeding manifestation scale according to previous report [24]. Twenty sex- and age-matched healthy controls (HC) were enrolled in the study. The study was approved by the Institutional Review Board of the hospital, and written informed consents were obtained from all participants.

### Metagenomic next-generation sequencing (mNGS)

Blood sample was drawn from ITP patient prior to the treatment, and plasma was obtained by centrifugation (1500*g*, 10 min). The process of mNGS for plasma pathogen detection consisted of nucleic acid extraction, library construction, sequencing, and bioinformatic analysis. Briefly, DNA was extracted from 0.3 ml plasma using a Nucleic Acid Purification kit (BGI Genomics, China) according to the manufacturer’s protocol. Then, DNA library was constructed by using an end-repair method. After polymerase chain reaction (PCR) amplification, the quality of DNA library was estimated by an Agilent 2100 Bioanalyzer (Agilent Technologies, USA). Qualified DNA library was sequenced on BGISEQ-2000 platform (BGI Genomics, China). The high-quality sequencing data was analyzed as described previously [25].

### Lipid extraction and lipid analysis

ITP patients and HC were asked to fast 12 h and rest for 20 min in a temperature-controlled room before drawing blood. Then, centrifugation to obtain plasma. Twenty microlitre plasma of each sample was used for lipid extraction. 9-lipid subclass Internal Standards kit were used for targeted quantitative detection. Nine subclasses include diacylglycerol (DAG), ceramide (CER), cholesteryl ester (CE), lysophosphatidylcholine (LPC), lysophosphatidylethanolamine (LPE), phosphatidylcholine (PC), sphingomyelin (SM), phosphatidylethanolamine (PE), and triacylglycerol (TAG). Samples were analyzed by liquid chromatography-mass spectrometry (LC–MS) on a Lipidyzer platform (QTRAP^®^ 5500, SCIEX, USA). Lipid peaks were identified by SCIEX OS (SCIEX, USA).

### Peripheral blood mononuclear cells culture

Peripheral blood mononuclear cells (PBMCs) were isolated from EDTA-anticoagulated venous blood using Ficoll density-gradient centrifugation, and cryopreserved in fetal bovine plasma (FBS) with 10% DMSO. Cryopreserved PBMCs were thawed at 37 °C water bath immediately, transferred to a 15 ml tube with Hank’s balanced salt solution (HBSS), and centrifuged at 450*g* for 5 min. The cells were resuspended in RPMI1640 supplemented with 10% FBS and seeded at 5 × 10^5^/mL in 24-well plates. For intracellular staining of interferon-γ (IFNγ), interleukin (IL)-4, IL-17, tumor necrosis factor-α (TNFα), transforming growth factor-β (TGFβ), Ki-67, and Granzyme B (GnB), cells were stimulated with Cell Stimulation Cocktail (eBioscience, USA) for 5 h.

### Magnetic isolation of CD4 T cells and CD4 T cell subsets culture

PBMCs were incubated with CD4 microbeads (Miltenyi, German) according to manufacturer’s instructions. CD4 T cells positive separations were performed by MiniMACS ™ Separator. CD4 T cells were resuspended in RPMI1640 supplemented with 10% FBS, 1 mM l-glutamine, 55 *u*M β-Mercaptoethanol, 200U/ml penicillin, and 100 *ug*/ml streptomycin. The cells were seeded at 5 × 10^5^/mL in 24-well plates which were precoated with 2 ug/ml anti-CD3 (BioGems, USA), and stimulated with 4 ug/ml anti-CD28 (BioGems, USA) and 10 ng/ml IL-2 (PeproTech, USA). IL-12 (50 ng/ml, PeproTech, USA) and anti-human IL-4 (10 ug/ml, PeproTech, USA) were used for T helper1 (Th1) culture. IL-4 (50 ng/ml, PeproTech, USA) and anti-human IFN-γ (10 ug/ml, PeproTech, USA) were used for Th2 culture. IL-6 (50 ng/ml, PeproTech, USA), IL-1β (10 ng/ml, PeproTech, USA), IL-23 (10 ng/ml, PeproTech, USA), TGF-β (10 ng/ml, PeproTech, USA), anti-human IFN-γ (10 ug/ml), and anti-human IL-4 (10 ug/ml) were used for Th17 culture. TGF-β (10 ng/ml), anti-human IFN-γ (10 ug/ml), and anti-human IL-4 (10 ug/ml) were used for regulatory T cells (Tregs) culture.

### Flow cytometry analysis

PBMCs were mixed gently with anti-CD4-FITC and anti-CD25-APC, and anti-CD8-APC antibodies (BD Pharmingen™, USA) respectively and incubated for 30 min at 4 °C, and washed twice. Then, fixed, permeabilized, and stained with anti-Foxp3-PE, anti-IFNγ-PE-CY7, anti-IL4-APC-CY7, anti-IL17-BV421, anti-TNFα-Percpcy5.5, anti-Ki67-FITC, anti-Granzyme B-PE, and anti-TGFβ-APC (BD Pharmingen™, USA) for analysis of T cells. The samples were performed by flow cytometry on a FACS Aric III flow cytometer (BD Biosciences, USA). Flow cytometry data were analyzed using FlowJo software 10.4.

### Mitochondrial reactive oxygen species and membrane potential measurement

MitoSOX ™ Red mitochondrial superoxide indicator (Invitrogen, USA) was used to measure cell mitochondrial reactive oxygen species (ROS) by flow cytometry after incubated at 37 °C for 20 min. The alteration of mitochondria membrane potential was measured by flow cytometry following staining with Enhanced mitochondrial membrane potential assay kit with JC-1 (Bytotime, China) in 37 °C incubator for 20 min. JC-1 forms aggregates emit red fluorescence while loss of membrane potential becomes monomers emit green fluorescence. The greater the accumulation of green fluorescence, the lesser the mitochondria membrane potential.

### Metabolism assays

Agilent Seahorse XF real-time ATP rate assay kit was used to measure OCR and ECAR using a Seahorse XFe96 Extracellular Flux Analyzer. T cells were plated at 2 × 10^5^ cell/well in assay media and stimulated following the injection of 1.5 uM oligomycin and 0.5 uM rotenone + antimycin A.

### Statistical analysis

The analysis was performed with GraphPad Prism 8.0 and SPSS 26.0. Data are expressed as mean ± standard deviation. Unpaired two-tailed student’s *t* test was used for comparisons between two groups and one way ANOVA were used for comparisons more than two groups. *P* value less than 0.05 was considered to indicate statistically significant.

## Results

### Patient characteristics and mNGS analysis

**Fig. 1:**
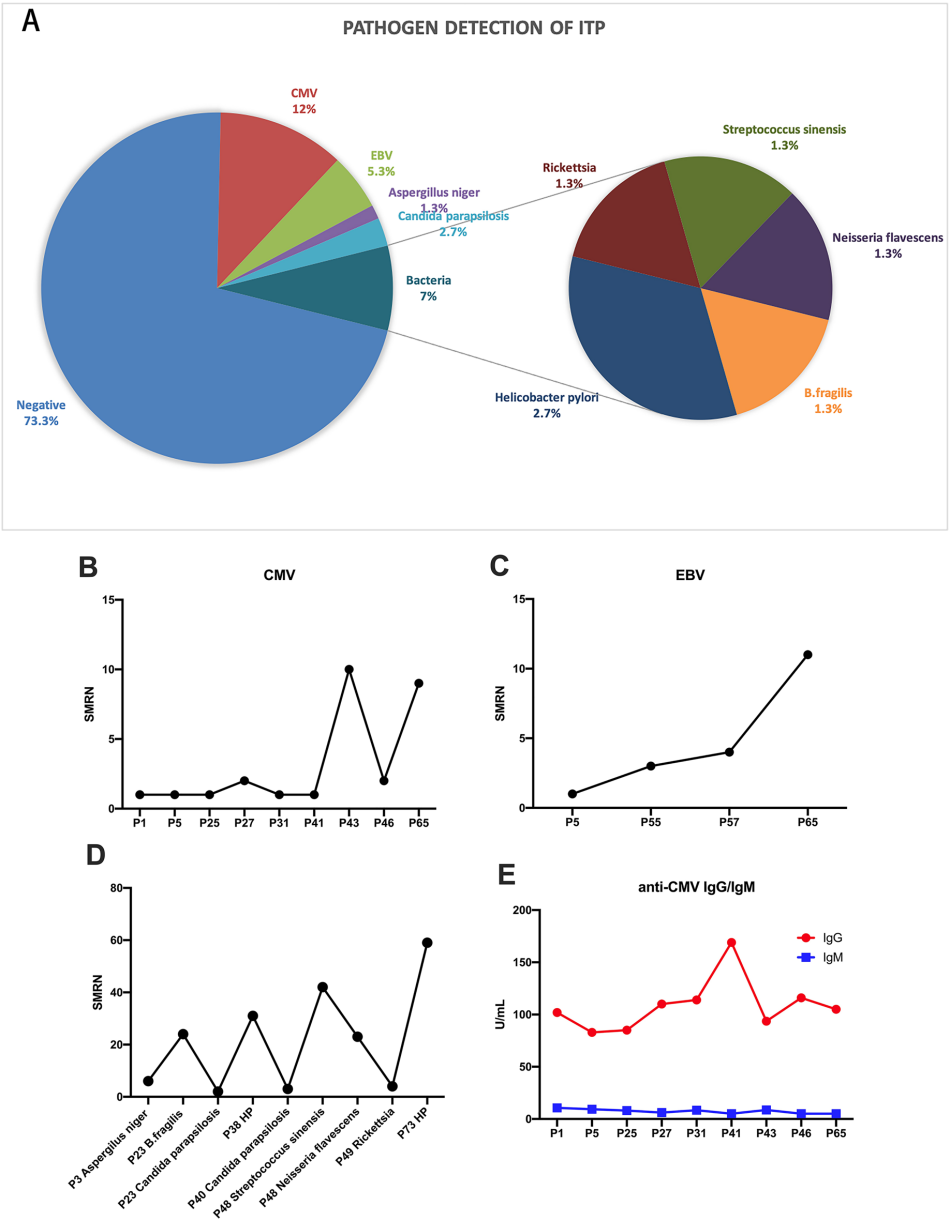
Metagenomic next-generation sequencing (mNGS) of ITP patients’ plasma.** A** Pie charts show the distribution of detected pathogens in ITP patients. The stringently mapped reads number (SMRN) of CMV (**B**), EBV (**C**) and other detected pathogens (**D**). **E** The expression of anti-CMV IgG antibodies and anti-CMV IgM antibodies. *ITP* immune thrombocytopenia, *CMV* cytomegalovirus, *EBV* Epstein-Barr virus

Seventy-five patients with newly diagnosed primary ITP with a median age of 53 years (range of 17–80 years) and a median platelet count of 11 × 10^9^/L (range of 1–33 × 10^9^/L) were enrolled to determine if they sustained with pathogen infection. Patients’ plasma was tested using mNGS. The clinical characteristics of the participants enrolled are shown in Table 1. As shown in Fig. 1A, the pathogen detection of plasma from ITP patients found 73.3% patients (55/75) were negative. Cytomegalovirus (CMV) were detected from 12% patients (9/75). Epstein-Barr virus (EBV) were detected from 5.3% patients (4/75). CMV and EBV were detected simultaneously in two patients. Aspergillusniger was detected from one patient and Candida parapsilosis was detected from two patients. Also, there were five kinds of bacteria were detected in the study, including 1 patient with B.fragilis, 2 patients with Helicobacter pylori, 1 patient with Richettsia, and 1 patient with Streptococcus sinensis and Neisseria flavescens. Candida parapsilosis and B.fragilis were detected simultaneously in 1 patient. Among the 7 patients accompany with bacterial or fungal infection, 71.4% (5/7) were NR after first-line treatment with corticosteroids.

**Table 1:** Clinical characteristics of study participants

Characteristics	ITP patients	Healthy controls
	(*n* = 75)	(*n* = 20)
Median age (range)	53 (17–80)	45 (26–70)
Gender		
Female	47	13
Male	28	7
Platelet count (× 10^9^/L), median (range)	11 (1–33)	243 (140–320)
Bleeding score at initial diagnosis, mean (range)	2.25 (1–5)	–
Response to corticosteroids		
CR + R	49	–
NR	26	–

*ITP* immune thrombocytopenia, *CR* complete response, *R* response, *NR* no response

### Stringently mapped reads number of pathogens and related antibodies

The levels of detected pathogens and their related antibodies expression were then analyzed. The stringently mapped reads number (SMRN) was used to evaluate the levels of detected pathogens. The SMRNs of detected pathogens were shown in Fig. 1B, C and D. The SMRN of CMV was 3.11 ± 3.66 (Fig. 1B) and EBV was 4.75 ± 4.35 (Fig. 1C). The SMRN of bacterium and fungus was 21.56 ± 19.98 (Fig. 1D). All of the 9 patients with CMV shown anti-CMV IgG antibodies were positive which above 14 U/mL (108.61 ± 8.52 U/mL). However, anti-CMV IgM antibodies were all negative which below 18 U/mL (7.36 ± 0.71 U/mL) (Fig. 1E). In addition, 4 patients with EBV by mNGS shown anti-EBVCA IgM and IgG antibodies were negative (data not shown). In summary of the results of SMRNs, antibodies, and literature [26], infections of CMV and EBV in ITP patients in the study were not considered to have the ability causing the disease.

### The change of lipid metabolic in ITP patients with positive antigens

**Fig. 2:**
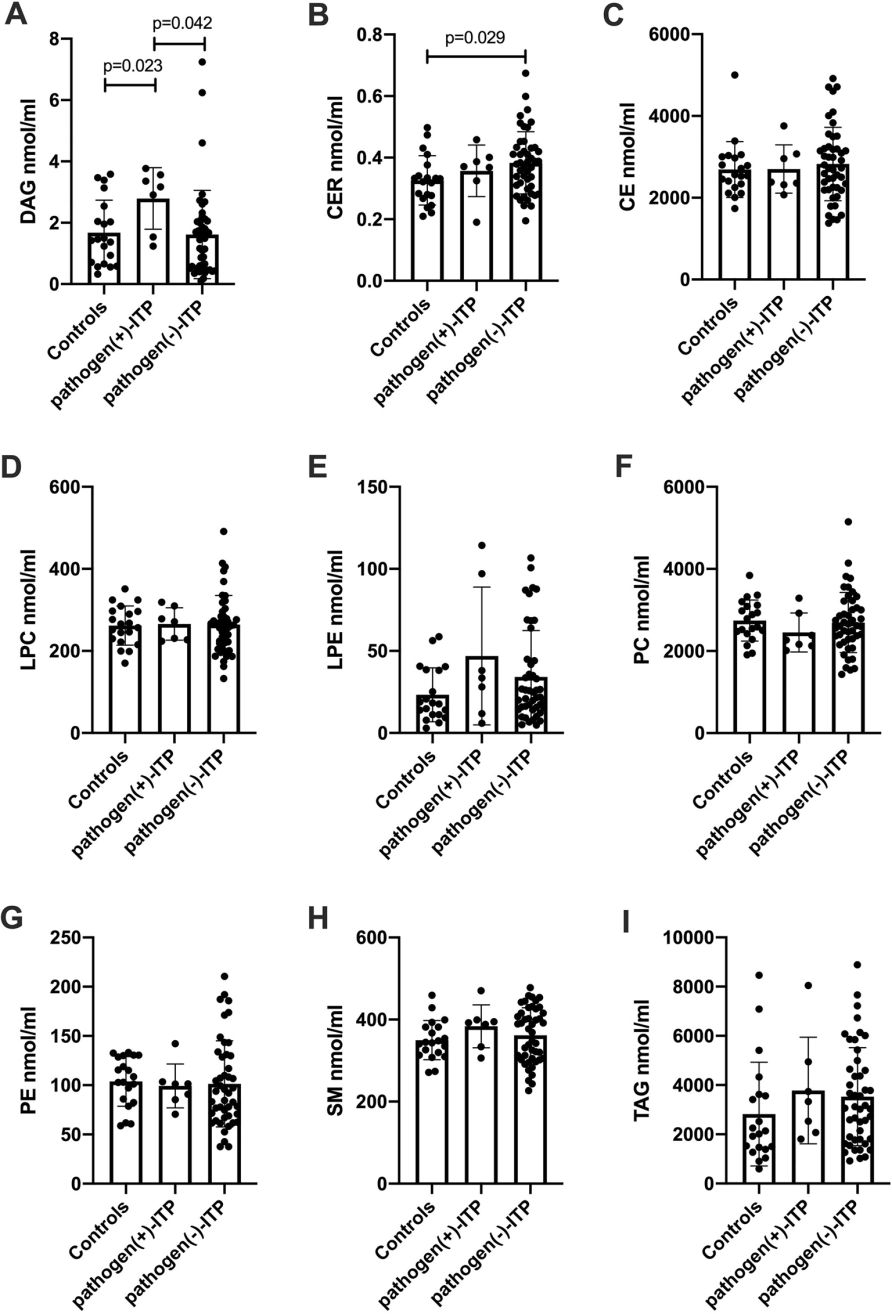
Plasma lipid subclasses from comparing pathogen( +)-ITP, pathogen(-)-ITP patients, and healthy controls. **A–I** The plasma levels of DAG, CER, CE, LPC, LPE, PC, PE, SM, and TAG in pathogen( +)-ITP, pathogen(−)-ITP patients, and healthy controls. Each bar graph represents mean ± SD. *P* values less than 0.05 were labeled in the figure. *ITP* immune thrombocytopenia, *DAG* diacylglycerol, *CER* ceramide, *CE* cholesteryl ester, *LPC* lysophosphatidylcholine, *LPE* lysophosphatidylethanolamine, *PC* phosphatidylcholine, *PE* phosphatidylethanolamine, *SM* sphingomyelin, *TAG* triacylglycerol

Furthermore, LC–MS was used to study whether ITP patients with positive antigens could cause the change of metabolic. Positive plasma pathogens were detected in 7 ITP patients in the study. Twenty volunteers were enrolled as healthy controls. As shown in Fig. 2, the level of DAG (Fig. 2A) was increased significantly in positive pathogen-ITP patients compared to HC and negative pathogen-ITP patients (*p* = 0.023, and *p* = 0.042, respectively). The level of CER (Fig. 2B) was increased significantly in negative pathogen-ITP patients compared to HC (*p* = 0.029). However, the level of CER was not significantly different between positive pathogen-ITP patients and HC or negative pathogen-ITP patients. Also, the levels of CE (Fig. 2C), LPC (Fig. 2D), LPE (Fig. 2E), PC (Fig. 2F), PE (Fig. 2G), SM (Fig. 2H), and TAG (Fig. 2I) were not significantly different among 3 groups.

### The changes of CD4 T cells in ITP patients with positive antigens

**Fig. 3:**
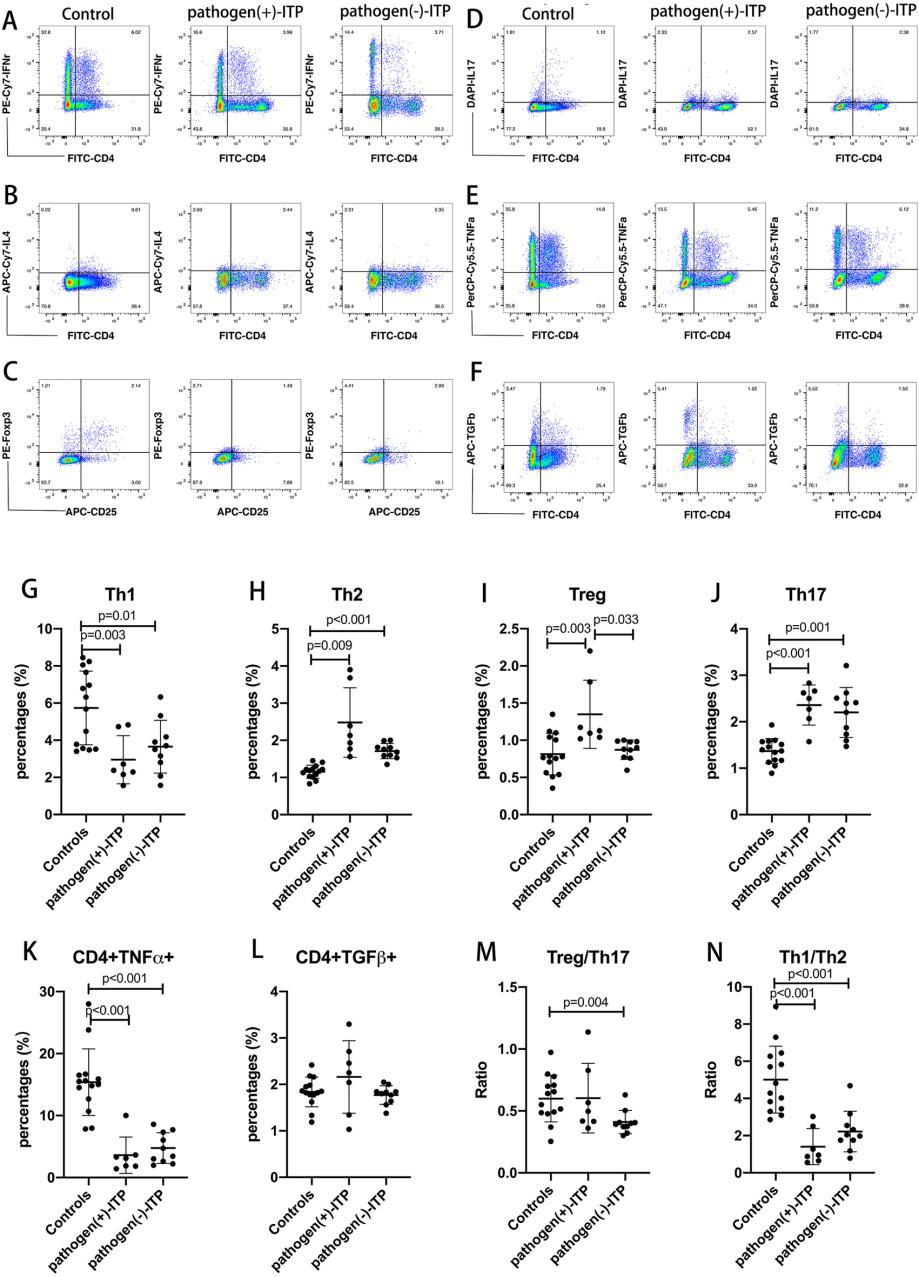
Flow cytometry analysis of Th1, Th2, Treg, Th17, CD4+ TNFα+ , CD4+ TGFβ+ cells in pathogen( +)-ITP, pathogen(-)-ITP patients, and healthy controls. **A–F** Representative dot plots of Th1, Th2, Tregs, Th17, CD4+ TNFα+ , and CD4+ TGFβ+ cells in the PBMCs of pathogen( +)-ITP, pathogen(−)-ITP patient, and healthy control. **G–N** The percentages of Th1, Th2, Tregs, Th17, CD4+ TNFα+ , and CD4+ TGFβ+ cells and the ratio of Treg/ Th17 and Th1/Th2 in the PBMCs of pathogen( +)-ITP, pathogen(-)-ITP patients, and healthy controls. Each bar graph represents mean ± SD. *P* values less than 0.05 were labeled in the figure. ITP, immune thrombocytopenia

As the immune system may be abnormally regulated by positive antigens, T cell subsets and function were evaluated for the changes of T cells in positive pathogen-ITP patients. Representative dot plots of Th1, Th2, Tregs, Th17, CD4 + TNFα + , and CD4 + TGFβ + cells in the PBMCs of HC, positive pathogen-ITP patient, and negative pathogen-ITP patient were shown in Fig. 3A, B, C, D, E and F. The percentages of Th1 cells (Fig. 3G) and CD4+ TNFα+ cells (Fig. 3K) were decreased significantly in positive pathogen-ITP and negative pathogen-ITP patients, as compared to HC (*p* < 0.05). The percentages of Th2 cells (Fig. 3H) and Th17 cells (Fig. 3J) were increased significantly in positive pathogen-ITP patients and negative pathogen-ITP patients, as compared to HC (*p* < 0.05). The percentages of Tregs were increased significantly in positive pathogen-ITP patients, comparing to negative pathogen-ITP patients and HC (*p* = 0.033 and 0.003, respectively) (Fig. 3I). The ratios of Treg/Th17 (Fig. 3M) and Th1/Th2 (Fig. 3N) were decreased significantly in negative pathogen-ITP patients than HC (*p* = 0.004, and *p* < 0.001, respectively). The ratio of Th1/Th2 was decreased significantly in positive pathogen-ITP patients as compared to that of HC (*p* < 0.001). However, there were no significant differences in the percentages of Th1, Th2, Th17, CD4+ TNFα+ , CD4+ TGFβ+ cells (Fig. 3L), Treg/Th17 ratio, and Th1/Th2 ratio between positive pathogen-ITP and negative pathogen-ITP patients.

### The changes of CD8 T cells in ITP patients with positive antigens

**Fig. 4:**
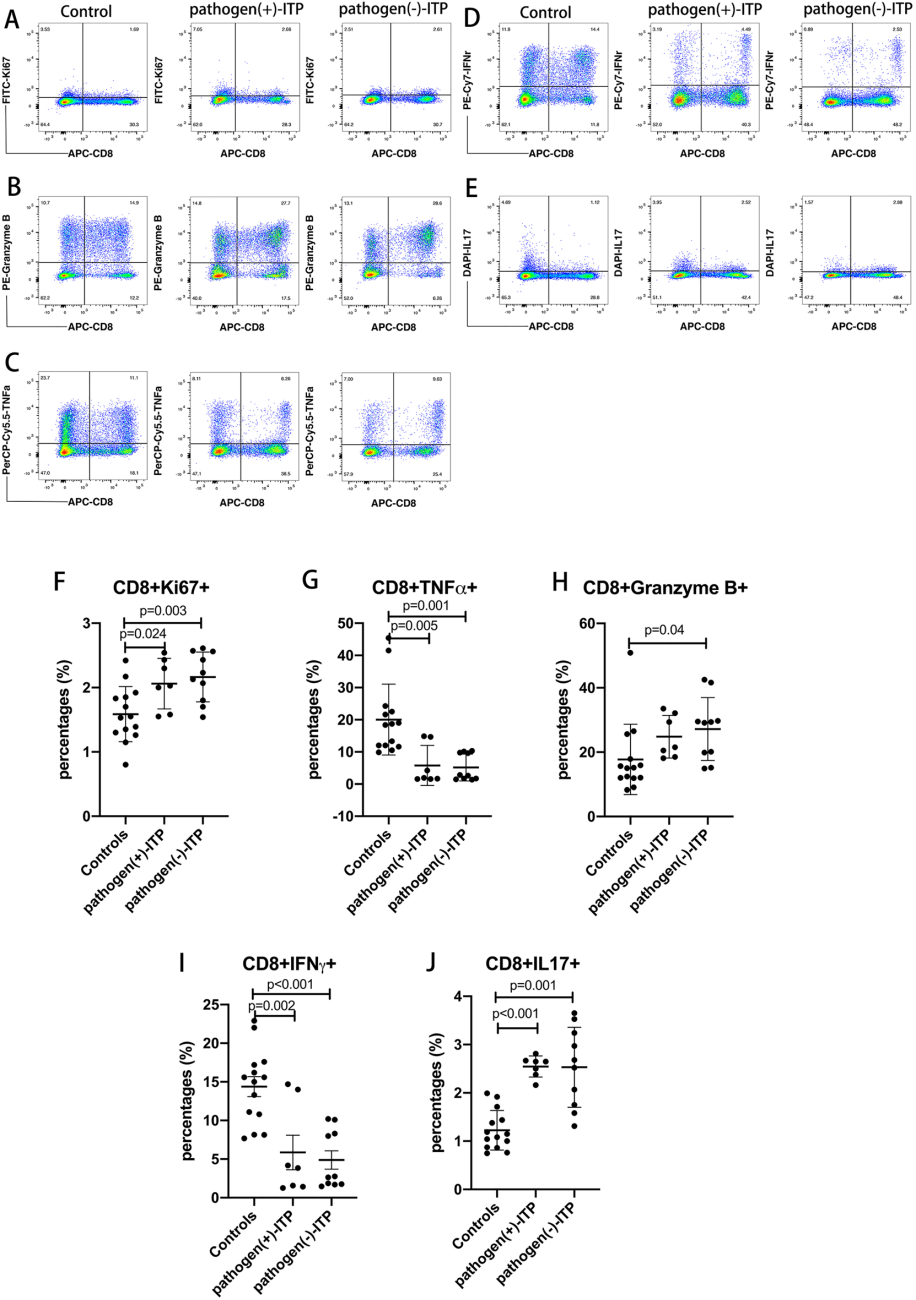
Flow cytometry analysis of CD8+ Ki67+ , CD8+ TNFα+ , CD8+ Granzyme B+ , CD8+ IFNγ+ , and CD8+ IL17+ cells in pathogen(+)-ITP, pathogen(−)-ITP patients, and healthy controls. **A–E** Representative dot plots of CD8+ Ki67+ , CD8+ Granzyme B+ , CD8+ TNFα+ , CD8 + IFNγ+ , and CD8+ IL17+ cells in the PBMCs of pathogen( +)-ITP, pathogen(-)-ITP patient, and healthy control. **F–J** The percentages of CD8+ Ki67+ , CD8+ TNFα+ , CD8+ Granzyme B+ , CD8+ IFNγ+ , and CD8+ IL17+ cells in the PBMCs of pathogen( +)-ITP, pathogen(−)-ITP patients, and healthy controls. Each bar graph represents mean ± SD. *P* values less than 0.05 were labeled in the figure. *ITP* immune thrombocytopenia

As shown in Fig. 4, representative dot plots of CD8+ Ki67+ , CD8+ GnB+ , CD8+ TNFα+ , CD8+ IFNγ+ , and CD8+ IL17+ cells in the PBMCs of HC, positive pathogen-ITP patient, and negative pathogen-ITP patient were shown in Fig. 4A, B, C, D and E. The percentages of CD8+ Ki67+ (Fig. 4F) and CD8+ IL17+ (Fig. 4J) cells were increased significantly in positive pathogen-ITP and negative pathogen-ITP patients than HC (*p* < 0.05). However, the percentages of CD8+ TNFα+ (Fig. 4G) and CD8 + IFNγ + (Fig. 4I) cells were decreased significantly in positive pathogen-ITP and negative pathogen-ITP patients than HC (*p* < 0.05). CD8+ GnB+ T cells (Fig. 4H) were increased significantly in negative pathogen-ITP than HC (*p* = 0.04). There were no significant differences in the percentages of CD8+ Ki67+ , CD8+ TNFα+ , CD8+ GnB+ , CD8+ IFNγ+ , and CD8+ IL17+ T cells between positive pathogen-ITP and negative pathogen-ITP patients.

### Changes of mitochondria membrane potential in ITP patients with positive antigens

**Fig. 5:**
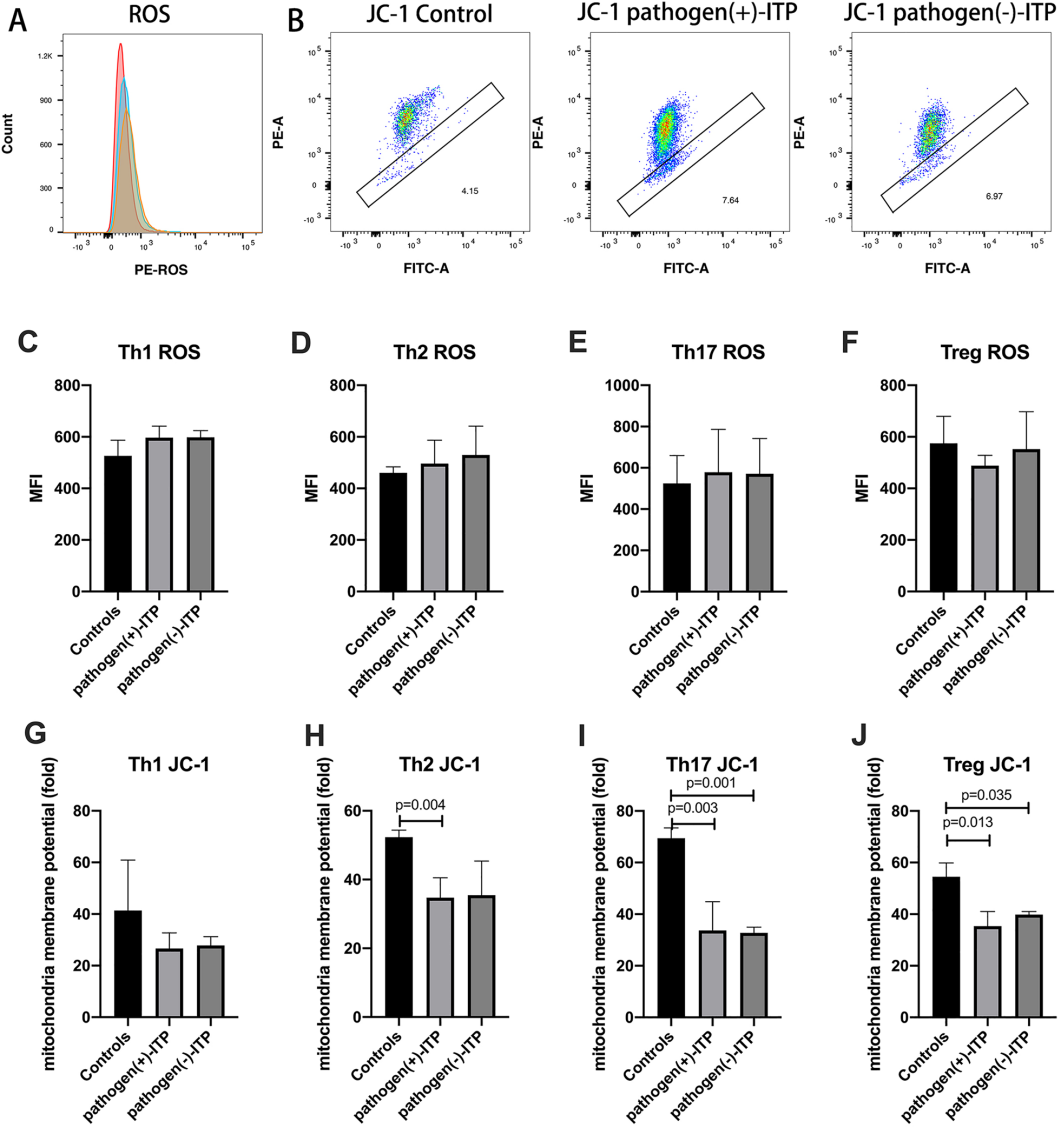
Flow cytometry analysis of Th1, Th2, Th17, and Treg mitochondrial ROS and mitochondria membrane potential in pathogen( +)-ITP, pathogen(−)-ITP patients, and healthy controls. **A**,** B** Representative plots of ROS and JC-1 in pathogen( +)-ITP, pathogen(−)-ITP patient, and healthy control. **C–F** The MFI of ROS of Th1, Th2, Th17, and Tregs in pathogen( +)-ITP, pathogen(−)-ITP patients, and healthy controls. **G–J** The mitochondria membrane potential of Th1, Th2, Th17, and Tregs in pathogen( +)-ITP, pathogen(−)-ITP patients, and healthy controls. JC-1 forms aggregates emit red fluorescence while loss of membrane potential becomes monomers emit green fluorescence. The greater the accumulation of green fluorescence, the lesser the mitochondria membrane potential. Each bar graph represents mean ± SD. *P* values less than 0.05 were labeled in the figure. *ITP* immune thrombocytopenia; ROS, reactive oxygen species

Considering the role of CD4 T cell subsets in ITP and the difference of Tregs in positive pathogen-ITP and negative pathogen ITP patients, researches on mitochondrial of CD4 T cell subsets were performed to investigate the changes of mitochondrial function in positive pathogen-ITP patients. Representative dot plots of ROS and JC-1 in control, positive pathogen-ITP patient, and negative pathogen-ITP patient were shown in Fig. 5A and B. The MFI of ROS in Th1 (Fig. 5C), Th2 (Fig. 5D), and Th17 (Fig. 5E) cells were increased in positive pathogen-ITP and negative pathogen-ITP patients compared to healthy controls but without significantly. The ROS of Tregs (Fig. 5F) was decreased in positive pathogen-ITP compared to negative pathogen-ITP patients and HC but without significantly. The result of mitochondria membrane potential shown that mitochondria membrane potential was decreased significantly in Th2 (Fig. 5H), Th17 (Fig. 5I), and Tregs (Fig. 5J) in positive pathogen-ITP patients compared to HC (*p* = 0.004, 0.003, and 0.013, respectively). Mitochondria membrane potential were decreased significantly in Th17 and Tregs in negative pathogen-ITP patients compared to HC (*p* = 0.001 and 0.035, respectively). There was no significant difference in mitochondria membrane potential between positive pathogen-ITP and negative pathogen-ITP patients.

### Suppressing OXPHOS in Tregs in ITP patients with positive antigens

**Fig. 6:**
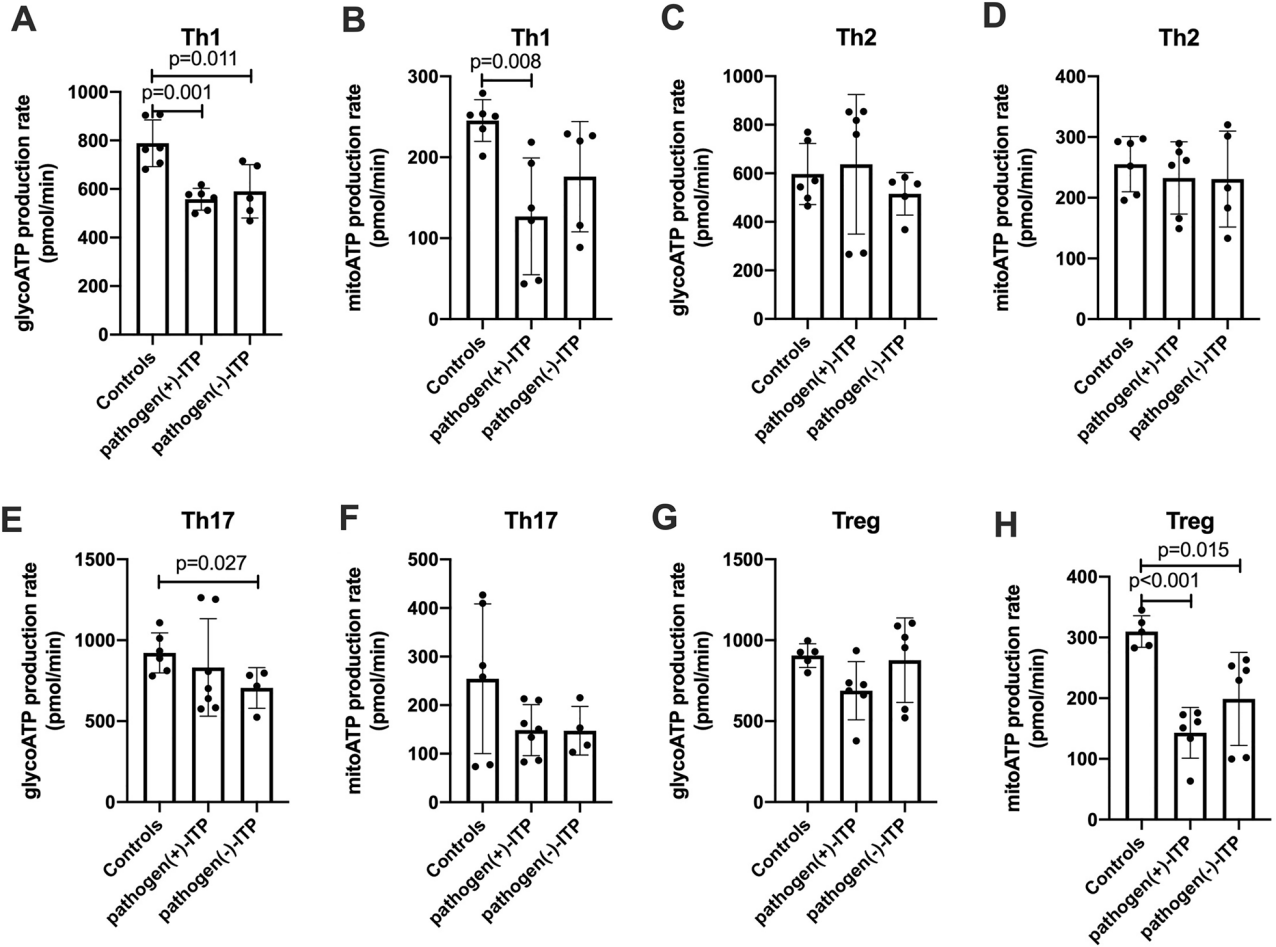
ATP production rate in Th1, Th2, Th17, and Treg in pathogen( +)-ITP, pathogen(−)-ITP patients, and healthy controls. **A**–**H** The production rate of glycolysis-derived ATP and mitochondrial-derived ATP of Th1, Th2, Th17, and Tregs in pathogen( +)-ITP, pathogen(−)-ITP patients, and healthy controls. Each bar graph represents mean ± SD. *P* values less than 0.05 were labeled in the figure. ITP, immune thrombocytopenia

Since mitochondria membrane potential were remarkably changed in positive pathogen-ITP patients and negative pathogen-ITP patients, the changes of mitochondria metabolism were tested. The glycolysis-derived ATP were decreased in Th1 cells in positive pathogen-ITP patients and negative pathogen-ITP patients compared to that of HC (*p* = 0.001 and 0.011, respectively) (Fig. 6A). The mitochondrial-derived ATP were also decreased in Th1 cells in positive pathogen-ITP patients than that of HC (*p* = 0.008) (Fig. 6B). The decrease of glycolysis-derived ATP in Th17 cells was shown in negative pathogen-ITP patients compared to HC (*p* = 0.027) (Fig. 6E). There were no significant differences in glycolysis-derived ATP in Th2 and Tregs among groups (Fig. 6C, G). Also, there were no significant differences in mitochondrial-derived ATP in Th2 and Th17 cells among groups (Fig. 6D, F). Compared to HC, the mitochondrial-derived ATP in Tregs in positive pathogen-ITP patients and negative pathogen-ITP patients were decreased (*p* < 0.001 and *p* = 0.015, respectively) (Fig. 6H).

**Fig. 7:**
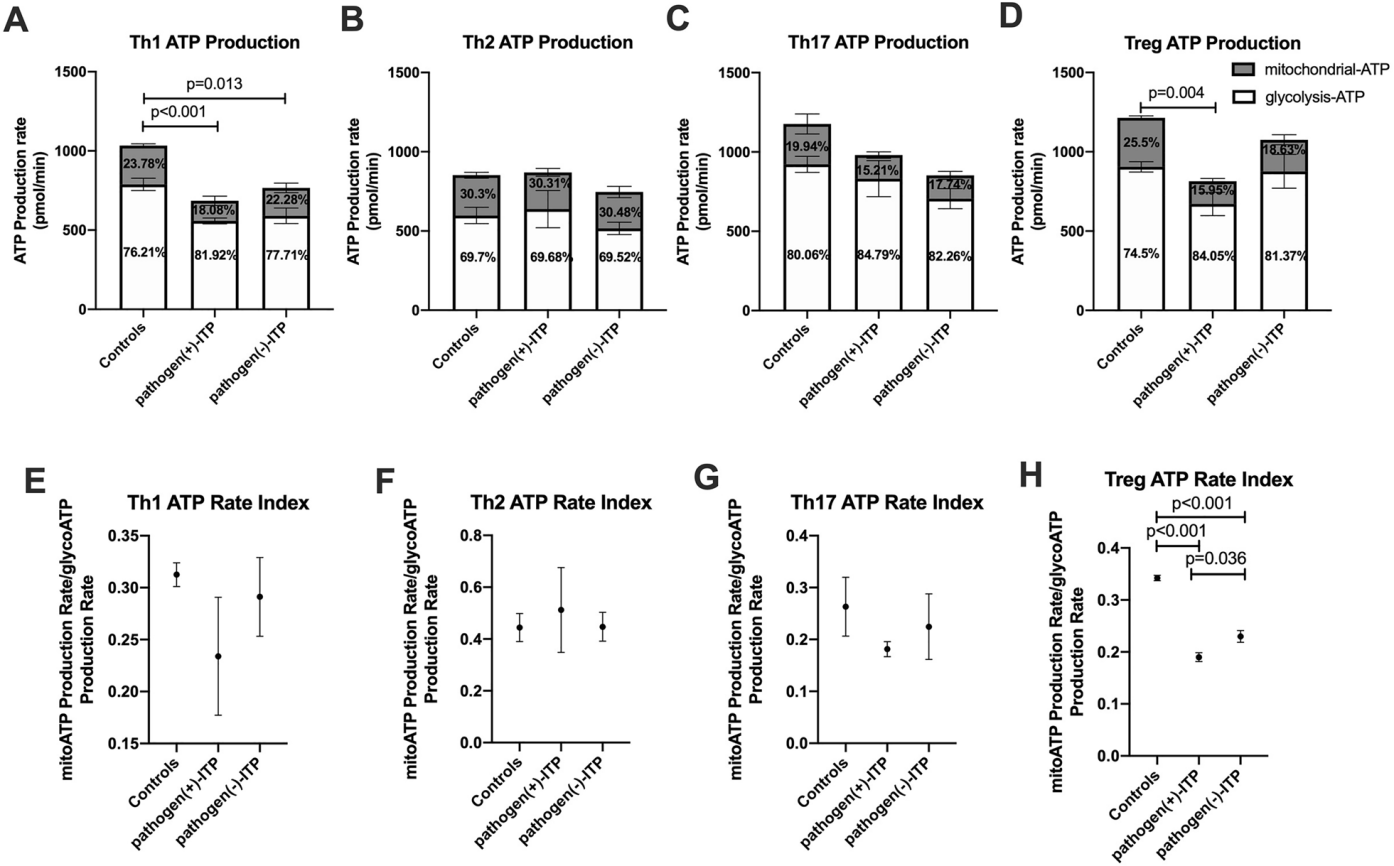
Seahorse XF real-time ATP rate analysis of Th1, Th2, Th17, and Treg in pathogen( +)-ITP, pathogen(−)-ITP patients, and healthy controls. **A–D** An overall metabolism flux of Th1, Th2, Th17, and Tregs in pathogen( +)-ITP, pathogen(−)-ITP patients, and healthy controls. (**E–H**) The ATP rate index of Th1, Th2, Th17, and Tregs in pathogen( +)-ITP, pathogen(−)-ITP patients, and healthy controls. Each bar graph represents mean ± SD. *P* values less than 0.05 were labeled in the figure. ITP, immune thrombocytopenia

The overall metabolism flux was decreased in Th1 and Tregs in positive pathogen-ITP patients compared to that of HC (*p* < 0.001 and *p* = 0.004, respectively) (Fig. 7A, D). The overall metabolism flux in Th1 cells of negative pathogen-ITP patients was decreased than that of HC (*p* = 0.013) (Fig. 7A). There were no significant differences in the overall metabolism flux in Th2 and Th17 cells among groups (Fig. 7B, C). The data showed that a smaller proportion of ATP was generated by OXPHOS in positive pathogen-ITP patients compared to negative pathogen-ITP patients and HC (15.95% versus 18.63% and 25.5%, respectively), and a higher proportion of ATP was generated by glycolysis compared to negative pathogen-ITP patients and HC (84.05% versus 81.37% and 74.5%, respectively) (Fig. 7D). The ATP rate index of mitochondrial-ATP production rate versus glycolysis-ATP production rate of Tregs was decreased significantly in positive pathogen-ITP and negative pathogen-ITP patients compared to HC (*p* < 0.001) (Fig. 7H). The ATP rate index of Tregs was decreased significantly in positive pathogen-ITP patients compared to negative pathogen-ITP patients (*p* = 0.036). There were no significant differences in ATP rate index of Th1, Th2, and Th17 cells among groups (Fig. 7E, F, G).

## Discussion

ITP is a highly heterogeneous disease. The individual differences of ITP patients may be related to the immune background conditions at the disease onset. ITP patients accompanied with pathogen infection is an established autoimmune manifestation. A study of 3440 ITP patients accompanied with HCV infection found a significant association between ITP development and HCV virus [27]. The potential mechanism of ITP development following pathogen infection is the molecular mimicry between pathogen component and human cells which lead to antigenic cross-reactivity [28, 29]. In present study, detected by mNGS, 26.7% of the ITP patients were found accompanied with viral or bacterial or fungal infection. The pathogenic microorganism detected by mNGS is the real pathogenic microorganism in the sample, which may be pathogenic microorganism, colonized microorganism, or background microorganism [25]. Of the patients with bacterial or fungal infection, 71.4% patients (5 out of 7) were NR after first-line treatment with corticosteroids, while 30.9% (21 out of 68) negative pathogen-ITP patients were NR after first-line treatment, strongly suggest that infection does affect treatment outcome.

T cells responding to pathogens or self-antibodies in autoimmune diseases acquire an altered state of differentiation to participate in immune response. CD4 T cell and CD8 T cell, including Th1, Th2, Th17, Tregs, and GnBCD8 T cells have been reported to have changes in quantity or function in autoimmune diseases, such as systemic lupus erythematosus (SLE), rheumatoid arthritis (RA), and ITP [6, 7, 22, 30, 31]. Our results are in line with these studies. In addition, current study showed that there were no significant differences in Th1, Th2, Th17, Th1/Th2 ratio, Treg/Th17 ratio, CD8 T cell, and most of the plasma lipid metabolites between positive pathogen-ITP and negative pathogen-ITP patients, indicating that the presence of microorganisms is not a driver to ITP pathogenesis and the pathogenesis of positive pathogen-ITP in present study are in accordance with primary ITP. T cells exhaustion, defined by the progressive loss of effector function after persistent infection or tumor antigens, lose the ability to produce cytokines, such as IFNγ, TNFα, and IL-2 [32, 33]. Interestingly, we found that IFNγ and TNFα in CD8 T cells and CD4 T cells were decreased significantly in positive pathogen-ITP and negative pathogen-ITP patients, indicating that T cell exhaustion possibly exist in ITP.

Pathogens cause diverse metabolic effects in different cell types [34, 35]. Glycolysis and mitochondrial OXPHOS are major pathways for energy generation and play key roles in energy homeostasis. Since glycolysis and mitochondrial OXPHOS are hubs for multiple metabolic pathways, diverse infectious pathogens take advantage of different pathways to their benefit. Glycolysis could be decreased or increased in response to pathogen infection. Dengue virus and herpes simplex virus 1 have been reported to activate glycolysis [36, 37]. Kaposi’s sarcoma-associated herpesvirus have been reported to cause a suppression of both OXPHOS and glycolysis [38]. T cells function has recently been linked to metabolism reprograms, which is considered as a checkpoint in immune response [15, 39]. Considering the role of CD4 T cell subsets in ITP and the difference of Tregs in positive pathogen-ITP patients and negative pathogen-ITP patients, the current study further investigated the mitochondrial function and cellular metabolism of CD4 T cell subsets. Mitochondria membrane potential was found to be decreased in Th2, Th17, and Treg in positive pathogen-ITP patients, revealing the impairment of mitochondria in positive pathogen-ITP patients. Furthermore, Tregs of positive pathogen-ITP patients were found to have a decreased mitochondrial-derived ATP and overall metabolism flux which was with a higher proportion of ATP generated by glycolysis and a smaller proportion of ATP generated by OXPHOS compared to that of negative pathogen-ITP patients and HC.

Tregs are well-known by their immune regulatory potential and play a vital role for maintaining immune homeostasis. In present study, glycolysis-derived ATP of Tregs were numerically decreased in positive pathogen-ITP patients compared to that of negative pathogen-ITP patients and HC, yet no statistic differences were found. Although both OXPHOS and glycolysis were suppressed in Tregs in positive pathogen-ITP patients, glycolysis seemed to have a more prominent role for ATP production. Taken together, results of current study revealed that impaired mitochondria of Tregs in positive pathogen-ITP patients caused the decrease of OXPHOS-derived ATP and an overall metabolism flux that could affect treatment outcome.

To date, this is one of the first attempts to evaluate infection in adults ITP patients using mNGS. A portion of ITP patients who have no obvious clinical symptoms of infection were detected with positive pathogens by mNGS. The T cell metabolism of these patients is remarkably different from that of ITP patients with negative pathogens. Abnormal T cell metabolism may be caused by some latent pathogens although there were no obvious clinical symptoms of infection. The study revealed that impaired mitochondria function of Tregs in positive pathogen-ITP patients cause the decrease of OXPHOS-derived ATP and an overall metabolism flux that could affect treatment outcome profoundly, which would be accounted for some refractory or recurrent situations. However, there are limitations in present study, such as small size of positive pathogen-ITP patients, and how the different pathogens cause mitochondrial dysfunction of Tregs and the mechanism in ITP patients remains unclear. As such further large scale, mechanistic investigations are warranted.

## Conclusion

Impaired mitochondria function of Tregs causes the decreases of OXPHOS-derived ATP and overall metabolism flux that might be the cause of steroid resistance in ITP patients with positive pathogen. For those ITP patients who do not respond well to first-line treatment with corticosteroids, analyzing for subclinical infections by mNGS would be helpful for subsequent treatment optimizing.

## Data Availability

All the data relevant to the study results are included in the main figures of the article or available in the additional files. The datasets used and/or analyzed during the current study are available from the corresponding author on reasonable request.

## Abbreviations

ITP: Immune thrombocytopenia
CR: Complete response
R: Response
NR: No response
mNGS: Metagenomic next-generation sequencing
Tregs: Regulatory T cells
CMV: Cytomegalovirus
EBV: Epstein-Barr virus
SMRN: Stringently mapped reads number
LC–MS: Liquid chromatography-mass spectrometry
DAG: Diacylglycerol
CER: Ceramide
CE: Cholesteryl ester
LPC: Lysophosphatidylcholine
LPE: Lysophosphatidylethanolamine
PC: Phosphatidylcholine
PE: Phosphatidylethanolamine
SM: Sphingomyelin
TAG: Triacylglycerol
ROS: Reactive oxygen species
GnB: Granzyme B
IFNγ: Interferon-γ
TNFα: Tumor necrosis factor-α
OXPHOS: Oxidative phosphorylation
